# Diversity and Distribution of Resistance Markers in *Pseudomonas aeruginosa* International High-Risk Clones

**DOI:** 10.3390/microorganisms9020359

**Published:** 2021-02-12

**Authors:** Béla Kocsis, Dániel Gulyás, Dóra Szabó

**Affiliations:** Institute of Medical Microbiology, Semmelweis University, 1089 Budapest, Hungary; gulyas.daniel@med.semmelweis-univ.hu (D.G.); szabo.dora@med.semmelweis-univ.hu (D.S.)

**Keywords:** *Pseudomonas aeruginosa*, multiresistance, nosocomial pathogen

## Abstract

*Pseudomonas aeruginosa* high-risk clones are disseminated worldwide and they are common causative agents of hospital-acquired infections. In this review, we will summarize available data of high-risk *P. aeruginosa* clones from confirmed outbreaks and based on whole-genome sequence data. Common feature of high-risk clones is the production of beta-lactamases and among metallo-beta-lactamases NDM, VIM and IMP types are widely disseminated in different sequence types (STs), by contrast FIM type has been reported in ST235 in Italy, whereas GIM type in ST111 in Germany. In the case of ST277, it is most frequently detected in Brazil and it carries a resistome linked to *bla*_SPM_. Colistin resistance develops among *P. aeruginosa* clones in a lesser extent compared to other resistance mechanisms, as ST235 strains remain mainly susceptible to colistin however, some reports described *mcr* positive *P. aeurigonsa* ST235. Transferable quinolone resistance determinants are detected in *P. aeruginosa* high-risk clones and *aac(6′)-Ib-cr* variant is the most frequently reported as this determinant is incorporated in integrons. Additionally, *qnrVC1* was recently detected in ST773 in Hungary and in ST175 in Spain. Continuous monitoring and surveillance programs are mandatory to track high-risk clones and to analyze emergence of novel clones as well as novel resistance determinants.

## 1. Introduction

*Pseudomonas aeruginosa* is a Gram-negative ubiquitous rod-shaped bacterium and one of the most common opportunistic human pathogens with the ability of causing a wide range of severe infections, including ventilator-associated pneumonia, otitis externa, burn wound infection, contact lense-related keratitis, and bloodstream infection [1,2,3,4]. Various antibiotic resistance mechanisms can occur in *P. aeruginosa* that enable it to develop multiresistance, therefore, it can cause difficult to treat infections. *P. aeruginosa* is a member of the “ESKAPE” group, and this group of pathogens comprises 6 multidrug resistant bacteria that are common nosocomial pathogens [5]. Moreover, infections caused by ESKAPE pathogens are usually associated with significantly high morbidity and mortality rates as limited number of effective antimicrobial agents are available against these pathogens [6,7,8,9].

Recently, the World Health Organization released a priority list for development of novel antibiotics against certain pathogens and carbapenem resistant *P. aerigunosa* is listed among ‘critical’ category pathogens, as research and development of antibacterial agents are urgently needed [10]. The need for novel antibiotics against *P. aeruginosa* is seen worldwide, as both intrinsic and acquired resistance mechanisms are detected in this pathogen [7,11,12].

*P. aeruginosa* exhibits intrinsic resistance to a variety of antibacterial agents due to its unusually decreased outer-membrane permeability as well as by upregulation of efflux pumps [13,14,15]. Furthermore, acquired resistance to beta-lactams and aminoglycosides can develop through acquisition of resistance genes [7,15,16]. The most common resistance to beta-lactams occurs through production of different beta-lactamases, namely AmpC beta-lactamases, extended-spectrum beta-lactamases (ESBLs), and carbapenemases [17,18,19,20]. The most frequently reported beta-lactamases in *P. aeuginosa* are ESBLs (e.g., PER, GES, VEB enzymes), OXA-type beta-lactamases, and metallo-beta-lactamases (MBLs) namely, IMP, VIM, NDM types [21,22]. Uncommon carbapenem resistance phenotype in *P. aeruginosa* has been also reported namely, carbapenem resistance with maintained cefalosporin susceptibility [23,24]. Aminoglycoside-modifying enzymes are also commonly detected in *P. aeruginosa* namely, *aacA4*, *aadA7*, *aph(3′)-IIb* [25,26,27]. Colistin resistance develops in *P. aeuginosa* either by alterations in two-component regulatory systems (PhoPQ and PmrAB) [28,29,30] or through acquisition of mobile genetic elements (*mcr*) [31]. Fluoroquinolone resistance develops by accumulation of mutations in gyrase and topoisomerase genes namely, in *gyrA*, *gyrB*, *parC*, and *parE* [32]. However, transfer of mobile genetic elements, such as integrons, integrative conjugative elements (ICEs), transposons, and plasmids play a role in dissemination of *crpP*, *aac(6′)-Ib-cr*, or *qnrVC1* fluoroquinolone resistance markers [20,33,34].

Emergence and worldwide dissemination of multidrug-resistant (MDR) *P. aeruginosa* made it necessary to apply typing methods for analysis of spread of MDR strains. Multilocus sequence typing (MLST) of MDR *P. aeruginosa* strains is the most common method to identify clones based on sequence type (ST) [35]. According to STs, high-risk clones are identified and their disseminations can be tracked. Additionally, core genome MLST has a higher resolution capacity that can be also applied in surveillance programs [36]. MDR clones are present worldwide and they evolve usually in hospital environments, as these clones are generally selected out in hospitals, due to antibiotic selection pressure and they can survive long-lasting in favorable conditions in hospital environments. Furthermore, genome of *P. aeruginosa* has a complexity and high variability, therefore *P. aeruginosa* can acquire and exchange resistance determinants that enables it for survival and dissemination. Thus, persistence of MDR *P. aeruginosa* empowers these strains for hospital outbreaks worldwide. These MDR *P. aeruginosa* are referred as international high-risk clones, such as the most frequently reported ST 111, 175, 233, 235, 277, 357, 654, and 773 [12,20,37,38]. In this comparative review, we analyze international high-risk clones based on their linkage and distribution of resistance determinants.

## 2. ST235

**Figure 1:**
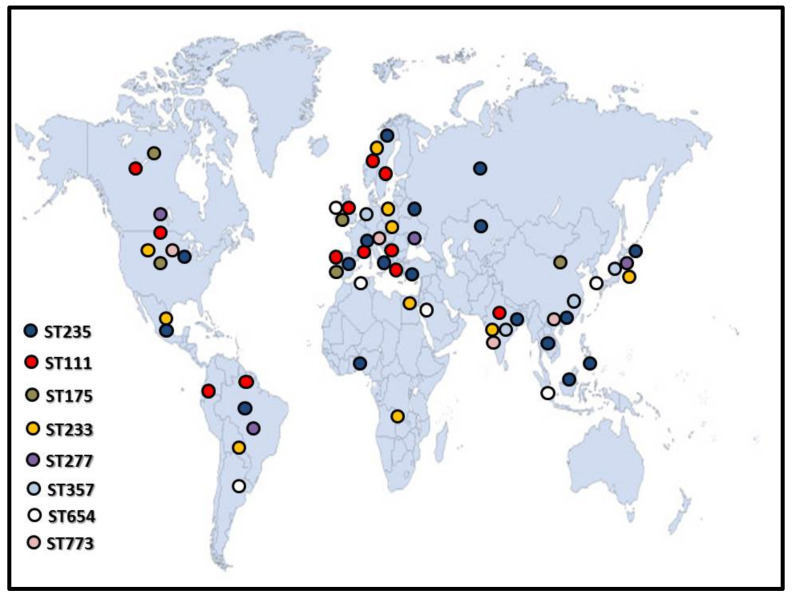
Geographic dissemination of the most frequently reported international high risk clones of *P. aeruginosa.* Figure illustrates reported cases until 2020. (source of map template: http://www.intrafor.com/locations-worldwide-presence.html, accessed on 23 January 2021).

According to all available data, this international high-risk clone is the predominant among sequence types in clinical isolates [37,38,39]. ST235 strains show a wide geographic distribution, as they were isolated in various countries, namely Belgium [40], Portugal [41], Hungary, Serbia [33], Greece, Italy, Croatia, Romania [12,42], Spain [43,44], France [45], Germany [46], Norway (this patient was hospitalized earlier in Cyprus) [47], and also from Korea [48,49], Japan [50], Nigeria [51], Brazil, Philippines, US, Mexico, India [12], Vietnam [52], Malaysia, Thailand [53], Russia, Belarus, and Kazakhstan [54] (Figure 1). 

The strains identified in ST235 clone carried various ESBLs, such as *bla*_PER-1_ in Serbia, and in Hungary [33], *bla*_PSE-1_ in Greece [12], *bla*_BEL-1_ in Belgium [40], chromosomally located class I integron (In1076) associated *bla*_GES-6_ in Portugal [41], class I integron co-harboring *bla*_GES-1_, *bla*_GES-5_ in Spain [43]. Various *bla*_OXA_ variants were found in strains of ST235, the most common one was *bla*_OXA-2_, but *bla*_OXA-1_, *bla*_OXA-10_, *bla*_OXA-11_, *bla*_OXA-35_, *bla*_OXA-74_, *bla*_OXA-17_, *bla*_OXA-50_, *bla*_OXA-129_ were also detected [12,51]. In the US, *bla*_PSE-1_ was detected however, in Mexico *bla*_GES-1_, *bla*_GES-19_, *bla*_GES-9-like_, whereas in Brazil, *bla*_CTXM-2_ was reported [12]. Additionally, *bla*_KPC-2_ was confirmed in samples from Colombia and Nigeria [12,55,56,57].

Vast majority of MBLs in strains of ST235 are IMP variants, namely *bla*_IMP-1_, *bla*_IMP-2_, *bla*_IMP-4_, *bla*_IMP-6_, *bla*_IMP-7_, *bla*_IMP-10_, *bla*_IMP-15_, *bla*_IMP-26_, *bla*_IMP-31_, *bla*_IMP-51_ from Malaysia, Korea, Philippines, Japan, Vietnam, and Nigeria [12,46,48,50,51,52,53]. Interestingly, *bla*_IMP-6_ and *bla*_IMP-10_ genes were reported as incorporated into genomic island (PAGI-16) in South Korea [49]. Among VIM types *bla*_VIM-1_, *bla*_VIM-2_, *bla*_VIM-4_*, bla*_VIM-11_, *bla*_VIM-13_, *bla*_VIM-47_ were reported [12,47,52,53,54,58,59]. Furthermore, *bla*_NDM-1_ carrier *P. aeruginosa* strains were isolated from a patient with acute pyelonephritis in France in 2012, and from a stem cell transplant recipient in Italy in 2013. Both patients were hospitalized previously in Serbia [11,60]. Subsequently, an another *bla*_NDM-1_ positive strain was reported in Vietnam in 2016 [52]. Later, in 2019, a *bla*_NDM-1_ positive *P. aeruginosa* was isolated in Italy from a patient who developed sepsis as a consequence of urinary tract infection caused by *P. aeruginosa* ST235. This strain carried different virulence determinants, including ExoU phospholipase that is an effector of type III secretion system and it is responsible for cytotoxic activity [61].

In *P. aeuginosa* ST235, a *bla*_FIM-1_ MBL gene was described in 2012 in Florence, Italy. FIM-1 beta-lactamase showed approximately 40% identity of amino acid sequence of NDM-type beta-lactamases. The *bla*_FIM-1_ was located on chromosome of *P. aeuriginosa* ST235 and flanked by ISCR19, that explains its insertion into the chromosome. However, mobilization and dissemination of this determinant is unknown. It seems that *bla*_FIM_ is endemic in Florence, as no distribution in other geographical regions has been recorded, yet [42].

In most cases, *aac(6’)-Ib* aminoglycoside acetyltransferase was reported in ST235 and it is responsible for resistance to kanamycin, tobramycin, and amikacin [12,51]. Strains of ST235 in Europe usually contained *aacA7* that encodes aminoglycoside 6’-N-acetyltransferase and some of them carried *aph(6)-Id* aminoglycoside O-phosphotransferase or *aadA1* adenylyltransferase. In case of plasmid-mediated quinolone resistance determinants, *aac(6′)-Ib-cr* was first described in *P. aeruginosa* ST235 in isolates from Serbia and Hungary [33].

Chloramphenicol resistance genes, *cmlA7* and *catB7* were also described in Hungary, Serbia, and Nigeria [33,52]. In a Nigerian sample, other resistance determinants were also confirmed, *fosA* for fosfomycin, *tetA*, *tetG* for tetracycline, and *qnrVC1* for fluoroquinolone resistance [52]. Compared to each other, strains of ST235 were characterized by quite similar resistance patterns however, they showed diversity based on the produced enzyme types and in the linkage to their geographic distribution.

A comprehensive study conducted in Madrid, revealed that MDR *P. aeuginosa* ST235 causes high mortality rate in patients in invasive infections, especially in patients with chronic respiratory tract infections [62].

## 3. ST111

This international successful clone is also responsible for numerous MDR *P. aeruginosa* cases around the world [63]. Reports from various European countries described ST111 in Portugal, Spain, Greece, France, Croatia, [12,64] Czech Republic [65], Sweden, and Norway [47]. (Figure 1) MDR ST111 isolates from Bulgarian hospitals showed a combination of ESBL production (VEB-1 and OXAs) with the lack of OprD carbapenem porin and overexpression of MexXY-OprM efflux pump resulting carbapenem resistance without harboring any encoded MBLs [66]. In The Netherlands, a nationwide emergence of this sequence type was also detected in 2012 [67]. The first full genome sequence of ST111 *P. aeruginosa* was described in the United Kingdom in 2014 and was deposited in the European Nucleotide Archive with accession number *PRJEB4573* [63] (Table 1).

ST111 strains were reported from Colombia [75], India, Venezuela, Canada, and United States [12]. In Columbia, a report detailed detection of co-carriage of *bla*_VIM-2_ and *bla*_KPC-2_ in a single *P. aeruginosa* strain that belonged to ST111. This strain was resistant to amikacin, ciprofloxacin, polymyxin, and to all beta-lactams, including carbapenems and aztreonam [75]. In 2015, almost 90 isolates of *P. aeruginosa* ST111 were analyzed in the United Kingdom. Most tested strains carried *bla*_VIM-2_, a few had *bla*_IMP-1_ or *bla*_IMP-13_, and one presented coexistence of *bla*_VIM-2_ and *bla*_IMP-18_, whereas another strain presented *bla*_NDM-1_ [68].

GIM type metallo-beta-lactamase was reported as an integron-encoded gene [76]. This MBL type shares 40% amino acid sequence similarity to IMP type MBLs. So far, *bla*_GIM_ was detected only in *P. aeruginosa* ST111, and seems endemic in Germany [77].

Apart from *bla*_VEB-1_, other ESBLs, such as *bla*_PSE-1_ and *bla*_GES-7_ were present in ST111 and numerous types of oxacillinases, such as *bla*_OXA-2_, *bla*_OXA-9_, *bla*_OXA-10_, *bla*_OXA-17_, *bla*_OXA-46_, *bla*_OXA-74_, *bla*_OXA-101_, *bla*_OXA-142_ were also described. Other resistance mechanisms, like *aac(6)-Ib* and *aacA29a* were the most common determinants of aminoglycoside resistance and amino acid substitutions in *gyrA*, *parC*, and *parE* were also confirmed in cases of fluoroquinolone resistant ST111 *P. aeruginosa* [12,64,66].

## 4. ST175

This epidemic high-risk clone was first identified in 2005 in the United Kingdom and Canada. Since then, it was reported in Hungary, the Czech Republic, Poland, the United States, China, Spain, and France [69,78]. (Figure 1) In case of ST175, *P. aeruginosa* infections the predominantly detected resistance mechanisms are associated to specific mutations in chromosomally encoded versatile resistome including *oprD* for inhibition of carbapenems, *ampR* for AmpC overexpression, *mexZ* efflux pumps, *gyrA* and *parC* as fluoroquinolone resistance markers [7,70,79]. Furthermore, presence of *bla*_CARB-12_, *catB7*, *bla*_IMP-1_, *aacA4′-3* in Japan [50], just as *bla*_VIM-2_, *bla*_IMP-22_, *aac(6)-Ib* in Spain were also confirmed associated to class I integron [78]. In a study conducted in Spain, a VIM-47 producing *P. aerugonosa* ST175 strain was reported [59].

In 2019, an MDR *P. aeruginosa* ST175 was identified in Spain. It carried *bla*_VIM-20_ and *bla*_OXA-681_ in class I integron on a plasmid. OXA-681 is a variant of OXA-2, and specific amino acid differences at position 159 and 160 distinguish the two beta-lactamases. OXA-681 is able to hydrolyze cephalosporins, including ceftolozane and induces cross-resistance to ceftolozane-tazobactam and ceftazidime-avibactam. Thereby it may cause serious clinical problems, because new combinations with antipseudomonal effect like ceftolozane-tazobactam and ceftazidime-avibactam are the most effective and recommended options for therapy of MDR *P. aeruginosa* infections. On the other hand, *bla*_OXA-681_ is located on a plasmid in a class I integron thus, it enables this resistance determinant for further dissemination. Gene accession numbers are available: ST175 genome sequence *SRX5389644*, *bla*_OXA-681_ MH986647.1 [69] (Table 1).

Recently, an interregional dissemination of ST175 MDR *P. aeruginosa* in Spain has been reported. Of the collected 33 strains, the majority carried *bla*_VIM-2_ however, *bla*_VIM-1_, *bla*_IMP-8_, *bla*_VIM-20_, *bla*_OXA-2_, *bla*_OXA-101_, *bla*_OXA-210_ were also detected. The coexisting resistance genes showed a wide variety, including *aac(6)-Ib*, *aac(6)-Ib-cr*, together with *aadA13*, *aadA1*, *bla*_TEM-1A_, *bla*_AER1_, *sul, qnrVC1*, and *catB7* [80].

## 5. ST233

Initially, ST233 was reported as a high-risk clone in Mexico, in the United States, in Japan, and in South Africa [81,82,83] (Figure 1). Earlier, this clone was described as colistin susceptible but in case of an extensively drug-resistant (XDR) strain resistance to colistin in 2013 was reported [81]. The first whole genome sequence of this clone was reported in Germany. The detected resistance genes *sul1*, *tet(G)*, *bla*_VIM-2_, *bla*_OXA-4_, *bla*_OXA-50_, *bla*_PAO_, *aac(3)-Id*, *aadA2*, *aph(3′)-IIb*, *dfrB5*, *fosA*, *catB7* were all located on bacterial chromosome. Assigned accession numbers *CP017293* and *CP017294* are added to GenBank (Table 1). In this study, the ST233 strains was exposed to colistin and colistin resistant strains were successfully selected through developing mutations in active genes contributing in lipopolysaccharide synthesis [71].

Whole genome sequencing was also performed on an XDR strain of ST233, called K34-7 in Norway. Its genome was built up by a chromosome and a small plasmid. The XDR K34-7 phenotype was encoded by resistance genes found in three class I integrons in chromosome, including *aac(3)*, *aac(3)-I*, *aac(6′)-Il*, *aadA2* and *aph(3′)-IIb, bla*_OXA-4_, *bla*_OXA-486_, *bla*_PDC-3_ and *bla*_VIM-2_, *catB*, *cmlA6*, and *floR*, *dfrB5*, *fosA*, *tetG* and as an accessoric feature, TetK tetracycline efflux pump was encoded on plasmid. This complete genome is available at GenBank under the accession numbers *CP029707* and *CP029708* [72].

Further studies demonstrated the presence of *bla*_IMP-1_ in Singapore [84], the copresence of *bla*_VIM-2_, *bla*_OXA-4_ with chromosomal mutations in *gyrA* and *parC* in Germany, France, Romania, US, and India [12]. Along with ST235 and ST111, *bla*_NDM-1_ was also detected in ST233 in tertiary care hospitals in Egypt in 2015 [85].

An interesting aspect of this clone is the confirmed case of reverse zoonotic effect in Brazil, called as zoo anthroponotic transmission. A dog was suffering from severe ear infection and by laboratory tests carbapenem-resistant ST233 *P. aeruginosa* was identified from this infected ear, oral, and rectal swab and later from the pet owner’s feces and also on the household settings. Interestingly, the pet owner, a 50-year-old man had 1-month long hospital stay in the ICU and after the successful recovery he was released from the hospital a month before the onset of the dog’s symptoms. This strain of ST233 carried chromosomally encoded beta-lactamases, namely *bla*_VIM-2_, *bla*_PAO_, *bla*_OXA-4_, *bla*_OXA-50_, and other different resistance determinants such as *aadA2*, *aac(3)-Id*, *aph(3)-IIb*), *sul-1*, *tet(G*), *fosA*, *catB7*, and *cmlA1*. These findings made attention for the colonization possibility in the household environments and further transmission to pets of hospital-acquired human pathogens, such as MDR *P. aeruginosa* [86].

## 6. ST277

Strains of ST277 are detected predominantly in Brazil from various clinical specimens. These strains have a quite characteristic resistance pattern that is remarkably restricted to the Brazilian area. These resistance markers comprise *bla*_SPM-1_, *bla*_OXA-56_, *bla*_PAO_, *rmtD*, *aacA4*, *aac(6)-Ib*, *aac(6)-Ib-cr*, *aph(3′)-IIb*, and *aadA7.* In certain cases, OXA-variants, like *bla*_OXA-396_ and *bla*_OXA-50_, as well as *fosA*, *sul-1*, *catB7* with chromosomal *gyrA* and *parC* mutations were also reported in strains of ST277. The related whole genome sequences are available under *MVOB00000000* and *JPSS00000000* accession numbers at GenBank [12,27,87] (Table 1).

Interestingly, scarce reports detail ST277 out of Brazil however, in the UK and Japan, ST277 has been already described. In Japan, this clone was identified with quite different genomic properties, it carried *bla*_IMP-1_, *bla*_IMP-10_, *aacA1* genes in urine samples [50]. In 2016, a SPM-1 producing *P. aeruginosa* ST277 that was only susceptible to colistin was reported in the UK from a patient who was hospitalized earlier in Brazil [88].

According to a study, which performed a phylogenetic analysis of almost fifty ST277 strains, the Brazilian isolates have a big number of single nucleotide polymorphisms and distinctively bear genomic islands (In163 and PAGI-25) compared to other ST277 genomes described in Asia, Europe, or North America. The presence of these special elements could explain the widespread dissemination of this endemic clone in Brazil [89].

Additionally, *P. aeruginosa* ST277 carried a 763,863 bp chromosomally coded unique region comprising two new genomic islands (PAGI-13 and PAGI-14), a mobile element, an integrative and conjugative element (ICE) associated to *bla*_SPM-1_. Furthermore, *rmtD* and In163 are inserted in PAGI-13 while PAGI-14 has genes encoding proteins related to type III restriction system and phages [87].

Strains of ST277 were isolated from microbiota of migratory birds in Brazil, suggesting that these birds might have played an important role in the widespread dissemination in this territory [38,90]. In this geographical region, the most commonly detected carbapenem resistance mechanism is *bla*_SPM-1_ (São Paulo metallo-β-lactamase) in clinical samples of MDR *P. aeruginosa*. Migratory bird-associated ST277 strains carried also *bla*_SPM_, and these strains were susceptible only to colistin and contained *aac(6′)-Ib-cr*, *rmtD1*, *aacA4* [90]. Furthermore, *P. aeruginosa* ST277 strains were also recovered from environmental water samples in Brazil [91].

## 7. ST357, ST654 and ST773

ST357 is also listed among high-riks clones and it is characterized by different *bla*_IMP_ determinants namely, *bla*_IMP-1_, *bla*_IMP-6_, *bla*_IMP-7_, *bla*_IMP-10_, *bla*_IMP-11_ [12,50,92,93]. Other beta-lactamases were described in ST357 as *bla*_OXA-2_, *bla*_OXA-4_, and *bla*_OXA-10_ [12,94], *bla*_VEB-1-like_ [12], *bla*_PER-1_ [52], *bla*_GES-5_, and *bla*_VIM-2_ [92]. Fosfomycin resistance encoding *fos1*, *fosE*, and a variety of aminoglycoside resistance genes such as *aadA11*, *A2*, *B*, *aacA3*, and *4*, *aph(3)-VIa*, *aac(6)-Ib*, *aph(6)-Id* were also described [12,52,92,93,94].

ST654 was also reported as a *bla*_IMP_ carrier, and *bla*_IMP-1_ and *bla*_IMP-26_ were reported from Singapore [84]. In Sweden, *bla*_VIM-2_ positive ST654 was reported in a patient who was hospitalized earlier in Tunisia [47]. Recently, VIM-producing *P.* *aeruginosa* ST654 strains were reported from a tertiary and quaternary hospital in Saudi Arabia [95]. ST654 was reported as *bla*_KPC-2_ positive in Argentina [96]. Furthermore, *bla*_NDM-1_ carriage was also reported in the UK [19].

In the case of ST773 *bla*_VIM-2_ and *bla*_OXA-10_ in India [53], the carriage of *bla*_VEB-1_ associated to *aac(3)-Ia*, *aadA1*, *aadB* in Vietnam [52], *bla*_OXA-181_ in the UK [19], *bla*_VIM-4_ and *bla*_IMP-2_ in Iran were reported [97].

However, recently, *bla*_NDM-1_ was also confirmed in ST773. This strain developed resistance to tobramycin, ciprofloxacin, and levofloxacin, and showed susceptibility to colistin. This strain of ST773 carried *qnrVC1* fluoroquinolone resistance marker localized on class I integron that was originated from *Vibrio cholerae*. Besides this resistance marker, that ST773 strain carried several other genes, such as *bla*_OXA-50-like_, *bla*_PAO_, *rmtB*, *aph(3′)-Iib*, *tetG*, *fosA*, *catB7*, and *sul1*. Whole genome sequence of this strain was deposited in GenBank under the accession number *RHDU00000000* [20]. A *bla*_NDM-1_ positive ST773 strain was also reported in USA from a patient who received medical care earlier in India due to her intraabdominal surgery. Whole genome sequence data are deposited at BioSample: *SAMN12307670* [74] (Table 1).

## 8. Discussion

Emergence and dissemination of *P. aeruginosa* international high-risk clones are great challenge worldwide [54,81,98]. MDR and XDR strains of high-risk *P. aeruginosa* are frequently identified in nosocomial infections and these cause difficult to treat infections as limited treatment options are available [60]. Furthermore, genome of *P. aeurginosa* has its complexitiy and high variability, therefore various resistance genes can be acquired from non-fermentative bacteria or even from different strains of Enterobacterales [74].

Development of MDR strain depends on given geographic area’s features, namely trends of antibiotic consumptions, patterns of transmittable resistant determinants among nosocomial pathogens, travelling habits, previous hospitalization events [11,47,60,87]. In addition, in several countries, such as Portugal, Spain, Romania, Italy, Croatia, Sweden, Norway, UK, US, India, Korea, and Japan, many clones (e.g., ST235) circulate with quite similar resistance pattern [12].

The most commonly detected *P. aeruginosa* clone is ST235 with a wide variety of beta-lactamases, including the most frequent *bla*_VIM-2_, *bla*_OXA-2_, *bla*_KPC-2_, *bla*_PAO_, and *bla*_NDM-1_. Moreover, *bla*_FIM-1_ was also reported in ST235 in Italy, however, this is the only detection of *bla*_FIM_ so far [42]. A few years ago, *bla*_NDM-1_ was the dominant MBL described in ST235, [11,52,60,61] but it has disseminated with an increasing tendency, since it was also detected in ST111 [68], ST233 [85], ST654 [19], and ST773 [20,74] (Table 2). Beside the most frequently isolated ST235, other sequence types have also important features. In ST175, OXA-681 was reported, which hydrolyzes ceftolozane and induces cross-resistance to ceftolozane-tazobactam and ceftazidime-avibactam. Possible transfer of *bla*_OXA-681_ contributes to diverse resistance pattern of *P. aeruginsa* and adds to the disquieting challenges related to antibiotic resistance [69].

ST235 and ST111 strains show mainly positivity for different ESBL production (*bla*_PES-1_, *bla*_BEL-1_, *bla*_GES_, *bla*_PER_), these genes are associated to *bla*_OXA_ variants and in certain cases to distinctive types of *bla*_VIM_ or *bla*_IMP_. Further resistance mechanisms against aminoglycosides, fluoroquinolones (mainly mutations in *gyrA* and *parC*), fosfomycin, tetracycline, and sulfonamides were also detected [12]. Recently, a study conducted in Greece reported predominance of VIM producing ST111 amd ST235. Interstingly, *bla*_VIM_ genes were incorporated into three integron types, namely, In59, In595, and In1760 [99]. 

The analyzed high-risk clones are worldwide disseminated, but as an exception, the ST277 is detected mostly in Brazil and shows a relevant spreading tendency in that geographical area [27,87]. Strains of ST277 carry distinct resistomes that are linked to *bla*_SPM-1_ and interestingly these strains are particularly colistin-sensitive. According to a study, Brazilian ST277 strains have several single nucleotid polymorphisms, and carry distinct genomic islands, phages, and CRISPR-Cas system compared to other ST277 genomes described in other countries, and these features could be the explanation for epidemic spread of ST277 in Brazil [27,87,89,100]. In strains of ST277, specific PAGIs were reported, that belong to ICEs. It must be underlined that ICEs play an important role in mobilization of carbapenemase genes [101]. It also turned out that migratory birds might have played an important role in transmission of ST277 in Brazil, furthermore, in environmental water sources, ST277 could be also isolated [90,91]. Additionally, other studies supported the zoonotic impact of MDR *P. aeruginosa* ST233, that these high-risk clones are able to be transmitted from human individuals to pets and the bacteria can circulate between them [86].

These features all underline the importance of possible exchange of resistance determinants and clones between animals, humans, environmental, and hospital source. Therefore, the “One health” approach seems the most accurate one to cope with antibiotic resistance.

As a summary: new antimicrobial agents and protocols for therapy are urgently needed to combat infections caused by MDR *P. aeruginosa*. Early detection and analysis by whole genome sequencing can help to follow evolutionary changes in MDR *P. aeruginosa*, to track dissemination of novel resistance determinants and emergence of international high-risk clones.

## Figures and Tables

**Table 1:** Whole genome sequences of high-risk *P. aeruginosa* clones from published reports.

Reference	Clone	Accession Number
Loconsole et al., 2020 [61]	ST235	*JAABOY000000000*
Tada et al., 2016 [52]	ST235, ST277	(SRA): *DRA003741*
Witney et al., 2014 [63]	ST111	*PRJEB4573*
Kos et al., 2015 [15]	ST111	*JUAZ00000000*
Turton et al., 2015 [68]	ST111	*ERS716506* to *ERS716591*
Arca-Suárez et al., 2019 [69]	ST175	*SRX5389644*
Cabot et al., 2016 [70]	ST175	*ERS1280254* to *ERS1280271* and *ERS128027*3 to *ERS1280276* (European Nucleotide Archive)
Dößelmann et al., 2017 [71]	ST233	*CP017293* and *CP017294*
Taiaroa et al., 2018 [72]	ST233	*CP029707 CP029708*
Silveira et al., 2014 [73]	ST277	*JPSS00000000*
Galetti et al., 2019 [27]	ST277	*MVOB00000000*
Kos et al., 2015 [15]	ST654	*JTYC00000000*
Kocsis et al., 2019 [20]	ST773	*RHDU00000000*
Khan et al., 2020 [74]	ST773	BioSample: *SAMN12307670*

**Table 2:** Overview of the most common resistance genes of high-risk *P. aeruginosa.*

The Most Common Clinically Relevant Resistance Genes in *P. aeruginosa*					
Clone	Beta-Lactamases	Aminoglycoside Modifying Enzymes	Resistance to Fluoroquinolones	Resistance to Colistin	Other
**ST235**	*bla*_PSE-1_, *bla*_BEL-1_, *bla*_PER-1_, *bla*_GES_-variants, *bla*_CTX-M_, *bla*_OXA-2_, *bla*_KPC-2_, *bla*_PAO_, *bla*_IMP_-variants, *bla*_VIM-2_ and variants, *bla*_FIM-1_, *bla*_NDM-1_	*aacA7*, *aph(6)-Id*, *aadA1*, *aac(6′)-Ib-cr*	*qnrVC1*, mutations in *gyrA* and *parC*	Strains susceptible to colistin. Sporadic reports of *mcr* carriage	*fosA*, *cmlA7*, *sul-1*, *tetA*, *tetGcatB7*,
**ST111**	*bla*_PSE-1_, *bla*_GES-7_, *bla*_VIM-2_, *bla*_KPC-2_, *bla*_IMP-1_ and variants, *bla*_VEB-1_, *bla*_OXA-2_, and variants *bla*_NDM-1_	*aac(6)-Ib*, *aacA29a*	mutations in *gyrA*, *parC*, or *parE*	Colistin-resistant strain is confirmed	*mexZ* efflux pump, *oprD*, *sul-1*, *catB7*
**ST175**	*bla*_CARB-12_, *bla*_IMP-1_, *bla*_VIM-2_, *bla*_TEM-1A_, *bla*_AER1_, *bla*_OXA-2_ variants, like *bla*_OXA-681_	*aac(6)-1b*, *aacA4′-3*	*qnrVC-1*, mutations in *gyrA* and *parC*	No data available	*oprD*, *ampR*, *mexZ* efflux pumps, *catB7*
**ST233**	*bla*_VIM-2_, *bla*_IMP-1_, *bla*_OXA-4_, *bla*_OXA-50_, *bla*_PDC-3_, *bla*_PAO_, *bla*_NDM-1_	*aac(3)-Id*, *aadA2*, *aph(3′)-IIb*	mutations in *gyrA* and *parC*	Colistin-resistant strain is confirmed	*dfrB5*, *fosA*, *sul1*, *tetG*, *catB7*, *cmlA1*
**ST277**	*bla*_SPM-1_, *bla*_IMP-1_, *bla*_OXA-56_, *bla*_PAO_	*aacA1*, *aacA4*, *aac(6)-Ib*, *aac(6)-Ib-cr*, *aph(3′)-IIb*, *aadA7*	mutations in *gyrA* and *parC*	No data available	*fosA*, *sul-1*, *catB7*
**ST357**	*bla*_PER-1_, *bla*_GES-5_, *bla*_LCR-1_, *bla*_IMP-1_ variants, *bla*_VIM-2_, *bla*_OXA-2_ variants, *bla*_VEB1_-like	*aadA11*, *aadA2*, *aadB*, *aacA3*, *aacA4*, *aph(3)-VIa*, *aac(6)-Ib*, *aph(6)-Id*	mutations in *gyrA* and *parC*	No data available	*fos1*, *fosE*, *sul-1*
**ST654**	*bla*_KPC-2_, *bla*_IMP-1_, *bla*_VIM-2_, *bla*_NDM-1_	*aacA4*, *aacA5*, *aadb*	mutations in *gyrA* and *parC*	No data available	*sul-1*
**ST773**	*bla*_OXA-50-_like variants, *bla*_VEB-1_, *bla*_IMP-2_, *bla*_PAO_, *bla*_VIM-2_, *bla*_NDM-1_	*rmtB*, *aph(3′)-Iib*, *aac(3)-Ia*, *aadA1*, *aadB*	*qnrVC1*, mutations in *gyrA* and *parC*	Strains susceptible only to colistin are detected	*fosA*, *sul-1*, *tetG*, *catB7*

## Data Availability

Not applicable.
